# Taiwan National Health Insurance and Proportional Physician Fee of Psychiatrist in General Hospital during the COVID-19 pandemic : Case Report

**DOI:** 10.1192/j.eurpsy.2023.2185

**Published:** 2023-07-19

**Authors:** S.-C. Wang, Y.-H. Lin

**Affiliations:** 1 Psychiatry, Tao Yuan General Hospital, Ministry of Health and Welfare, Taiwan, Taoyuan City, Taiwan, Province of China

## Abstract

**Introduction:**

In Taiwan, National Health Insurance has been implemented for 27 years and continues to receive international recognition. People pay part of the quota at the time of medical treatment, and the rest of the medical expenses will be paid by the national health insurance. In this study, the researcher, a psychiatrist in the general hospital, investigated the correlation between service and revenue. He has started to work in this hospital since November 1st, 2021, without any other psychiatrist peers.

**Objectives:**

This study used proportion of PPF as performance indicator and aimed to observe the changes of PPF unit from November 1st, 2021, to January 31st, 2022, examining the trend of PPF growth. The purpose is to figure out an appropriate model to optimize medical services and performance outcomes.

**Methods:**

Demographic data were collected through PPF projects, consisting of 17 inpatient ward items and 14 outpatient items from November 1st, 2021, to January 31st, 2022, and items with no performance or related to physiological examination has been excluded. In addition, items with a ratio of greater than 1.5% are presented in the bar graphs, as shown in **Figure 2 and 3**. The performance proportion of inpatient ward and outpatient were calculated separately.

**Results:**

Demographic data found PPF rises significantly over time **(Figure 1)**. The 2nd month PPF unit (27.09%) was 2.5 times the 1st month PPF unit (10.70%), and the 3rd month PPF unit (62.21%) was 2.2 times the 2nd month PPF unit (27.085%). The highest proportion of PPF items were general hospital bed inpatient consultation fee for inpatient ward item **(Figure 2)** and psychiatric outpatient consultation fee for outpatient item **(Figure 3)**. Furthermore, only the proportion of psychiatric outpatient consultation – more than two consecutive transfers increased continuously and the proportion of psychiatric outpatient consultation – adjusted by the hospital decreased, the other items has not changed significantly.

**Image:**

**Figure:**
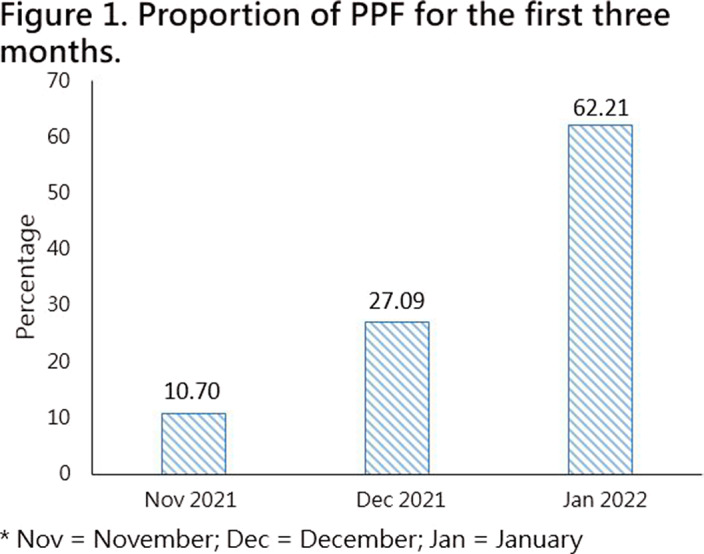

**Image 2:**

**Figure:**
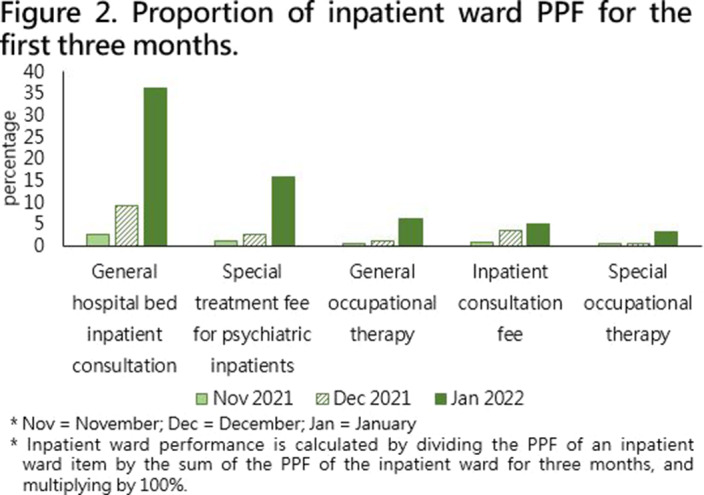

**Image 3:**

**Figure:**
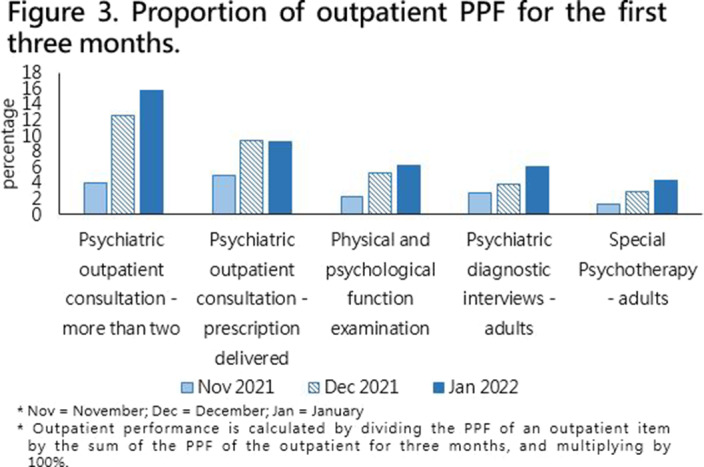

**Conclusions:**

In the first three months of the psychiatrist employment, the performance showed an increasing trend. These findings may suggest that the psychiatrist could be competent in a general hospital with patients’ confidence. In addition, under an optimal model of PPF and medical service, psychiatrists would more like to work in the general hospital, to serve acute psychiatric patients in need.

**Disclosure of Interest:**

None Declared

